# Extraordinary fossils reveal the nature of Cambrian life: a commentary on Whittington (1975) ‘The enigmatic animal *Opabinia regalis*, Middle Cambrian, Burgess Shale, British Columbia’

**DOI:** 10.1098/rstb.2014.0313

**Published:** 2015-04-19

**Authors:** Derek E. G. Briggs

**Affiliations:** Department of Geology and Geophysics and Yale Peabody Museum of Natural History, Yale University, PO Box 208109, New Haven, CT 06520-8109, USA

**Keywords:** Burgess Shale, *Opabinia*, Cambrian explosion, fossil preservation

## Abstract

Harry Whittington's 1975 monograph on *Opabinia* was the first to highlight how some of the Burgess Shale animals differ markedly from those that populate today's oceans. Categorized by Stephen J. Gould as a ‘weird wonder’ (*Wonderful life*, 1989) *Opabinia*, together with other unusual Burgess Shale fossils, stimulated ongoing debates about the early evolution of the major animal groups and the nature of the Cambrian explosion. The subsequent discovery of a number of other exceptionally preserved fossil faunas of Cambrian and early Ordovician age has significantly augmented the information available on this critical interval in the history of life. Although *Opabinia* initially defied assignment to any group of modern animals, it is now interpreted as lying below anomalocaridids on the stem leading to the living arthropods. This commentary was written to celebrate the 350th anniversary of the journal *Philosophical Transactions of the Royal Society*.

## 1. Introduction

*Opabinia* is one of the more unusual looking Cambrian animals, with its long anterior proboscis, five eyes on top of the head, elongate body with wide swimming appendages and tail with overlapping flaps. Whittington's redescription of *Opabinia* heralded the presence of very unusual creatures among those under investigation from the Burgess Shale, a deposit in which, remarkably, soft-bodied forms were fossilized [1]. It led to a resurgence of interest in the origins of biodiversity in the oceans and the relationships between the various animal groups then and now. Research activity on the explosion of diversity during the Cambrian, and the forms fossilized in such exceptional preservations, has increased ever since.

## 2. Whittington the trilobite expert

Harry Whittington (1916–2010) [2–4] started his research career in 1936 as a PhD student at Birmingham University in the UK mapping Lower Palaeozoic rocks in the Berwyn Hills of North Wales and investigating their contained fossils. He spent time at Yale University as a Commonwealth Fellow before taking up a lectureship in Judson College, Rangoon in 1940. Whittington returned to Birmingham as a lecturer in 1945 at the end of the Second World War and resumed fieldwork on the Ordovician rocks around Bala in Wales. Four years later, however, he was offered a post at the Museum of Comparative Zoology at Harvard University where he focused on trilobites, particularly examples that had been silicified during fossilization and could be extracted by dissolving the limestones that contained them and viewed in the round. Apart from their value as a source of new trilobites, Whittington's silicified specimens proved an incomparable repository of data on stages of development–trilobite ontogeny. While at Harvard, Whittington became the *de facto* international authority on trilobites, one of the most widely known groups of invertebrate fossils, publishing monographs on material from North America and the UK. Trilobites are abundant owing to their robust mineralized dorsal skeletons and because, like all arthropods, they grew by moulting, so one individual had the potential to generate multiple fossils. However, their soft parts, the non-mineralized cuticle of the limbs for example, are rarely preserved. Understanding the ventral morphology of trilobites required the evidence of exceptionally preserved specimens and some of the best examples of appendages, those of the Middle Cambrian trilobite that we now know as *Olenoides serratus*, had been illustrated by Charles Walcott in 1918 in one of his publications on the fossils from the Burgess Shale [5].

## 3. Burgess Shale beginnings

Walcott discovered the Burgess Shale in 1909 and published a series of descriptions of the fossils he collected in the *Smithsonian Miscellaneous Collections*. Walcott described his work as preliminary but, even following his death in 1927, there was relatively little research activity on the specimens [6]. The bulk of Walcott's collection in the National Museum of Natural History was relatively inaccessible, and there had been no further collecting of any consequence of Burgess Shale fossils, apart from a small amount of material amassed by an expedition led by Percy Raymond of Harvard University in 1930. This collection was housed in the Museum of Comparative Zoology in the large room in which Whittington worked. It had been curated by Ian Rolfe, subsequently Keeper of Geology at the Hunterian Museum, University of Glasgow, when he worked as a postdoc at Harvard in the early 1960s under Whittington's direction [7]. Rolfe also published on the Harvard material of the Burgess Shale fossil *Proboscicaris*, now known to be part of the anomalocaridid *Hurdia*, at this time [8]. When Armin Öpik, Estonian palaeontologist then working in Australia, visited Harvard in the 1960s his remark to Whittington that Raymond's fossils should be restudied apparently struck a chord [3]. In addition to having Burgess Shale specimens in cabinets in the room he occupied, Whittington had reviewed Walcott's collection in the National Museum of Natural History in Washington so, through a number of avenues, he was well aware of the potential importance of these Cambrian fossils.

In the mid-1960s, the Geological Survey of Canada (GSC) embarked on a project to map the area of Yoho National Park, including Walcott's Quarry, and Whittington [9] indicated that he was interested in coordinating a new investigation of the Burgess Shale. In due course, he was invited to spearhead this effort by his old friend Digby McLaren, who subsequently became the Director of the GSC. The GSC mounted two collecting expeditions, in 1966 and 1967, on which Whittington, accompanied by his wife Dorothy, played a major role. The excavation was carefully monitored, and the levels at which the specimens were found were recorded, so that associations between taxa could be determined. The specimens yielded by these labours ended up in Ottawa, finally establishing a Canadian collection, whereas Walcott's huge accumulation was in Washington and Raymond's much smaller one at Harvard. There is no evidence that Whittington had a preconceived idea of what the new Burgess Shale investigation would yield—his aim, presumably, was to improve our understanding of the morphology and taxonomy of the fossils. The prevailing wisdom, represented by Leif Størmer's treatment in the high profile *Treatise on invertebrate paleontology*, which grouped the Burgess Shale arthropods alongside trilobites in Trilobitomorpha, was that their appendages at least were trilobite-like [10]. Whittington, the trilobite expert, may not have expected his redescriptions to yield anything particularly remarkable.

## 4. The route to *Opabinia*

Before Whittington had even completed his Burgess Shale fieldwork, he had accepted an offer to succeed Oliver Bulman FRS in the Woodwardian Chair at the University of Cambridge. Thus, the nascent Burgess Shale project crossed the Atlantic with its leader who was returning to his English origins but with a rather different research focus to the silicified trilobites that, *inter alia*, had attracted him to Harvard 17 years earlier. Whittington's expertise and focus was arthropods, and he started his work redescribing the Burgess Shale fauna with *Marrella* [11], which, coincidentally, Walcott had named for John Marr FRS who held the Woodwardian Chair from 1917 to 1930. *Marrella* is the most abundant arthropod in the Burgess Shale by a significant margin, and Whittington used it to work out the mode of preservation of the Burgess Shale fossils showing that they, albeit flattened, retain some three-dimensionality and are oriented in a variety of attitudes to bedding owing to the way they were transported prior to burial [12].

Abundance may have been a criterion in determining how Whittington prioritized taxa for reinvestigation, at least initially (although some of the more abundant arthropods were investigated by his collaborators Christopher Hughes and David Bruton, and I, as a graduate student, was assigned the bivalved forms). Thus, the next arthropod that Whittington [13] tackled was *Yohoia*, which differed from *Marrella* particularly in the possession of a pair of grasping appendages (which Whittington referred to as the anterior great appendage) at the front. A second species of *Yohoia* described by Walcott turned out to lack these appendages and had a bivalved carapace, so Whittington assigned it to a new genus which he named *Plenocaris*. He investigated the limbs of the trilobite *Olenoides* [14] for the first international trilobite conference in Oslo in July 1973, although the papers from that meeting were not published until 1975. At the same time, he had embarked on his research on *Opabinia*. When Whittington showed a preliminary reconstruction at the Palaeontological Association conference in Oxford in 1972, the audience responded with loud and spontaneous laughter [1,6], an indication of how unfamiliar the creatures of the Cambrian appeared 40 years ago. My recollection is that Whittington was surprised by the reaction—he was not accustomed to having his work generate amusement! In addition to appearing somewhat strange, *Opabinia* is relatively rare, so it is not clear why it attracted Whittington's attention so early in the sequence of Burgess Shale papers. Such decisions were made well in advance; specimens had to be borrowed and mailed across the Atlantic. But there is no sign that Whittington changed the order in which he planned to research the taxa from the Burgess Shale in response to any development in his overview of the fauna. I doubt that he could have predicted that *Opabinia* would become a talisman for the Burgess Shale project as a whole.

**Figure 1.**
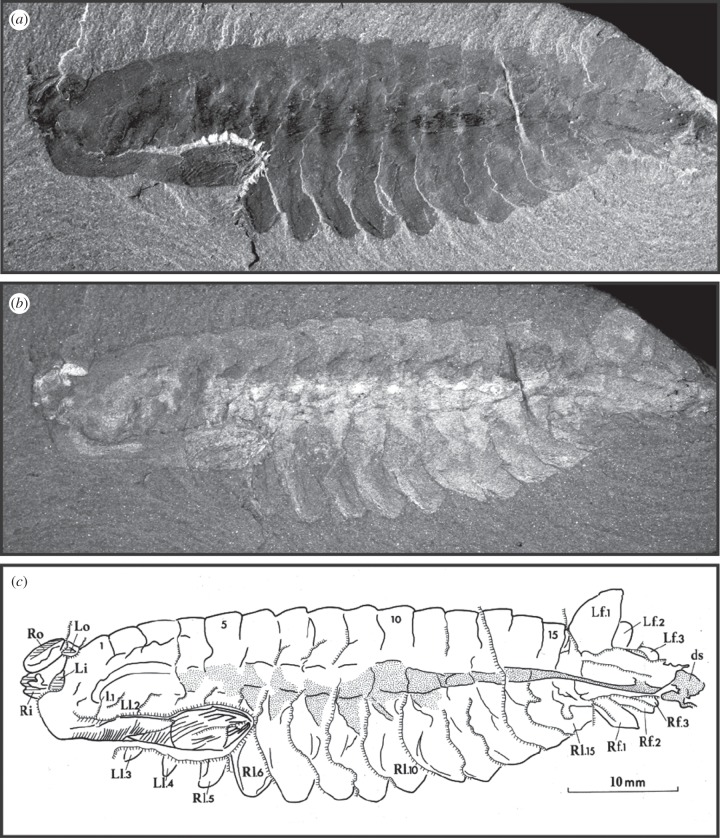
*Opabinia regalis* Walcott, 1912; USNM 155600 preserved in lateral view. (*a*,*b*) Counterpart ‘flipped’ horizontally to match (*c*), Whittington's [1, fig. 35] composite explanatory drawing of the part and counterpart; (*a*) is illuminated from a low angle and (*b*) from a high angle to generate reflection in the eyes, for example, showing the method Whittington used to illustrate different features of the Burgess Shale specimens. The needle-marks evident in (*a*) show where Whittington prepared the specimen to reveal the terminal spines on the ‘flexible frontal process’ or proboscis which is flexed backwards underneath the body. (*c*) Whittington's camera lucida drawing and interpretation. L and R indicate features on the left and right sides of the body; i and o, inner and outer eyes; l, lateral lobes of the trunk, numbered from the anterior; f, blades of the tail fan; ds, dark stain representing material that has ‘leaked’ beyond the body.

Whittington's *Opabinia* paper [1] is a comprehensive monographic description. His approach, with this and other Burgess Shale animals, was to review all the specimens, including those previously described (Walcott's original description was published in 1912 [15]), and select the most informative (figure 1). He used a modified dental drill to prepare specimens in order to reveal features obscured by matrix or even by other parts of the animal [9]. Whittington took photographs of the fossils under ultraviolet light, illuminating them from different angles to emphasize particular features. Most importantly, perhaps, he set a new standard by making detailed explanatory drawings, sometimes incorporating information from both part and counterpart, that served to inform and illustrate his interpretations [9]. He achieved this using a camera lucida, an apparatus with prism and mirror that fits on a binocular microscope and projects an image of the specimen onto a sheet of paper where it can be traced. These drawings were published, as far as possible, alongside his photographs providing a clear explanation of the evidence for his interpretations. Whittington described 10 specimens of *Opabinia* in detail—eight from the Smithsonian Institution National Museum of Natural History and one each from the Yale Peabody Museum and the GSC. All except the last, which was found during the GSC excavation of 1967, were collected by Walcott's expeditions. Whittington had access to a further 18 specimens from the Smithsonian collections.

Whittington's papers on *Marrella* and *Yohoia* appeared in the *Bulletin of the Geological Survey of Canada*; the original plan was that the Survey would publish the results of the reinvestigation of the Burgess Shale. The *Opabinia* monograph was the first of 12 descriptions of Burgess Shale animals to be published in the *Philosophical Transactions* by Whittington and his colleagues; others were published in other journals. This shift in publication strategy marked a recognition that the output of what was, by then, a team working under Whittington's guidance, would be too much for the Survey publication programme. Whittington's 1971 election to the Fellowship of the Royal Society may also have contributed to the change—the *Philosophical Transactions* became more accessible to him as a vehicle for monograph-length treatments.

## 5. The context—earlier research

Whittington's was not the first, nor the last, technical description of *Opabinia*. The morphology and affinities of this animal have generated significant controversy. Walcott's preliminary description was based on four specimens, two of which he figured [15]. He regarded *Opabinia* as a crustacean and compared it with the living anostracan branchiopod family Thamnocephalinae, which have large clasping antennae [15]. He conceded, however, that the specimens showed no trace of the characteristic appendages of the crustacean head—antennules, antennae, mandibles or maxillae—and he had difficulty in reconciling the linear structures on the lobes (which Whittington interpreted as gills) with the morphology of the trunk limbs of branchiopods. Walcott [15] noted that the elongate many-segmented body, leaf-like swimming appendages with an insignificant or rudimentary walking branch and small head of *Opabinia* are ‘very suggestive of an annelidan ancestor’.

**Figure 2.**
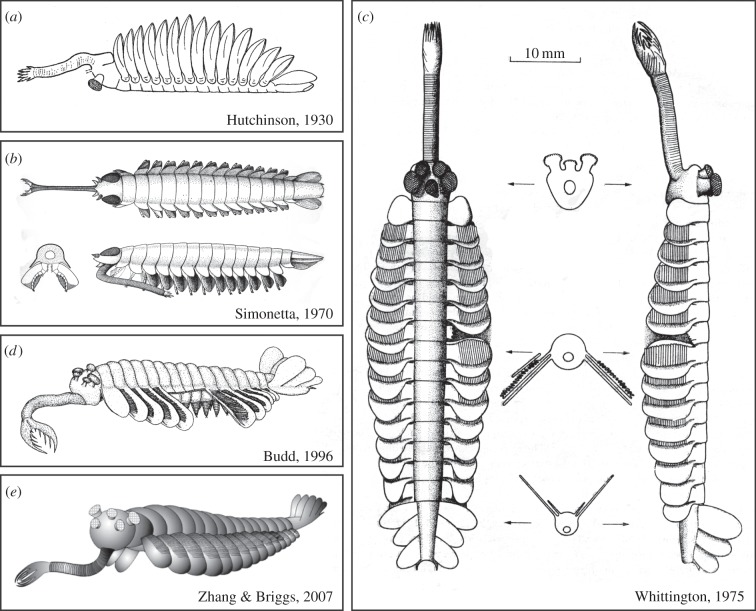
Major published reconstructions of *Opabinia regalis* Walcott, 1912 through time. Whittington's [1, fig. 82] version shows a dorsal and lateral view with the lobe and gill of segment 7 removed to show those of segment 8 in full. The position of the three cross sections of the body, with the outline of the gut internally, are indicated by the arrows. ((*d*) and (*e*) reproduced from [34] and [35] respectively with permission from John Wiley and Sons.)

The great ecologist G. Evelyn Hutchinson considered both *Opabinia* and the lobopod *Aysheaia* early in his career, in a paper published in 1930, shortly after he joined Yale University. Hutchinson [16] accepted Walcott's interpretation of *Opabinia* as an anostracan branchiopod and suggested that the proboscis might represent the fused internal branches of the antennae. He interpreted the trunk limbs as foliaceous with setae. Hutchinson provided the first reconstruction of *Opabinia*, showing the animal swimming ventral side up in the manner of a living anostracan (figure 2*a*). He regarded *Opabinia* [16, p. 10] as being ‘considerably less generalized than the modern Anostraca’. He erected a new suborder Palaeanostraca to accommodate *Opabinia* and other extinct taxa, which he interpreted as early specialized marine anostracans predating the migration of the group to fresh water. Hutchinson illustrated two specimens of *Opabinia* from the Smithsonian collections that were not figured in Walcott's original description. Both were figured and described by Whittington [1].

Alberto Simonetta, a professor of zoology at the University of Camerino and primarily a specialist on birds, was the first to recognize that Walcott's fossils merited a major reinvestigation. He began a comprehensive review of the arthropods in the Smithsonian collection in 1960, in part with his colleague Laura Delle Cave. Unlike Whittington and his team, however, Simonetta did not prepare the fossils, indeed, he may not have been permitted to do so. His description of *Opabinia* [17] was included in his fourth paper on the non-trilobite arthropods from the Burgess Shale. In it, he figured one of Walcott's original specimens and three others. Simonetta's 1970 paper also treated *Leanchoilia* and *Yohoia*, and his reconstruction of *Opabinia* (figure 2*b*) is reminiscent of those great appendage arthropods and very different from Hutchinson's anostracan-like version. Simonetta [17] depicted *Opabinia* with a bifid proboscis, short antenna-like appendages, just two large compound eyes, a dorsal exoskeleton with lateral projections (pleurae) and biramous trunk appendages with a long segmented inner branch. Whittington [1, p. 40] remarked that Simonetta's ‘restoration embodies features for which I find no evidence’, including antennae and jaws, eyes, biramous appendages and the nature of the tail fan. Gould [6, p. 131; see also 18] described Whittington ‘dissecting’ the specimens of *Opabinia* in search of the jointed limbs that should surely have been present if it were an arthropod. While the specimens show only limited evidence of preparation (figure 1*a*), Whittington's deep understanding of the way the Burgess Shale fossils split into part and counterpart ensured that he knew where to look for any potential segmented endopod and was confident they were absent.

## 6. No place for *Opabinia* among modern forms

Having drawn and interpreted the most instructive specimens of *Opabinia*, Whittington generated a reconstruction that looked quite different from those of Hutchinson and Simonetta, and did not closely resemble any living or fossil arthropod (figure 2*c*). It was clear that *Opabinia* did not belong with the trilobites (in Trilobitomorpha) nor could it be accommodated in any modern taxonomic group. To what was this strange creature related? It would probably be fair to say that he was flummoxed! Whittington turned for advice on the affinities and mode of life of *Opabinia* to one of the leading authorities of the day on living arthropods, Sidnie Manton FRS (1902–1979) of Queen Mary College and the Department of Zoology at the Natural History Museum (then British Museum (Natural History)) London, who was writing her 1977 textbook *The Arthropoda: habits, functional morphology and evolution* and was, in turn, relying on Whittington for advice on fossil arthropods [19]. On 6 March 1974, Whittington wrote to Manton ‘I don't think it is an arthropod—no jointed legs'. Her reply on 15 March includes a ‘fairy tale about *Opabinia*’ (clearly implying uncertainty in her mind about its nature) in which she suggests that *Opabinia* is ‘an annelid worm of an extinct group’. Whittington's letter to Manton on 22 April 1974 asks ‘is there any reason why it should necessarily be annelid in affinities, or should I merely suggest it is an extinct worm-like creature?’ (correspondence in archives of Sedgwick Museum of Earth Sciences, University of Cambridge, ref.: WHTN Box 33). When Whittington finally submitted his paper to the *Philosophical Transactions* on 6 August 1974, he noted [1, p. 41] that ‘this enigmatic animal thus exhibits features common to arthropods and annelids, but cannot be placed in any recognized group of either’. At that time, in contrast to our modern molecular-based understanding of the relationships of the major animal groups, annelids and arthropods were thought to be related.

**Figure 3.**
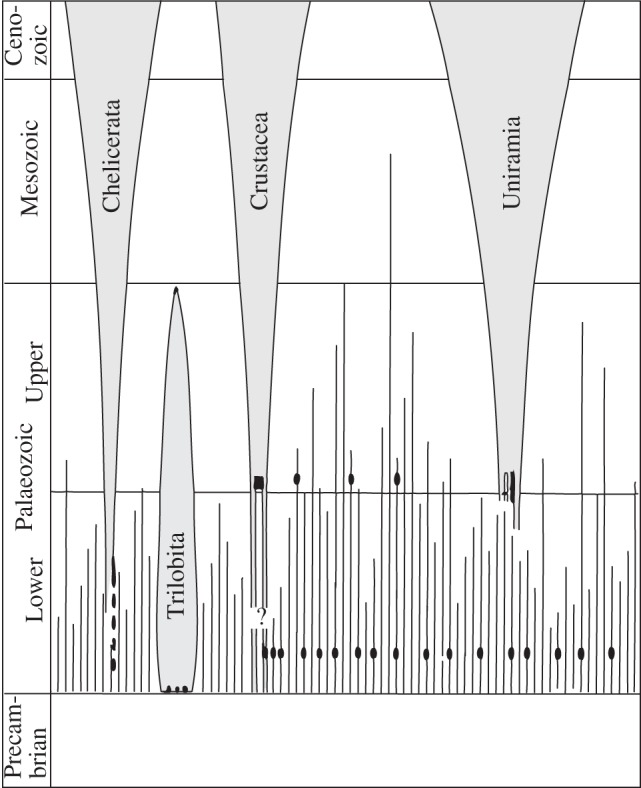
Whittington's (1979) diagram of the pattern of evolution in arthropods (adapted from [22, fig. 2]).

**Figure 4.**
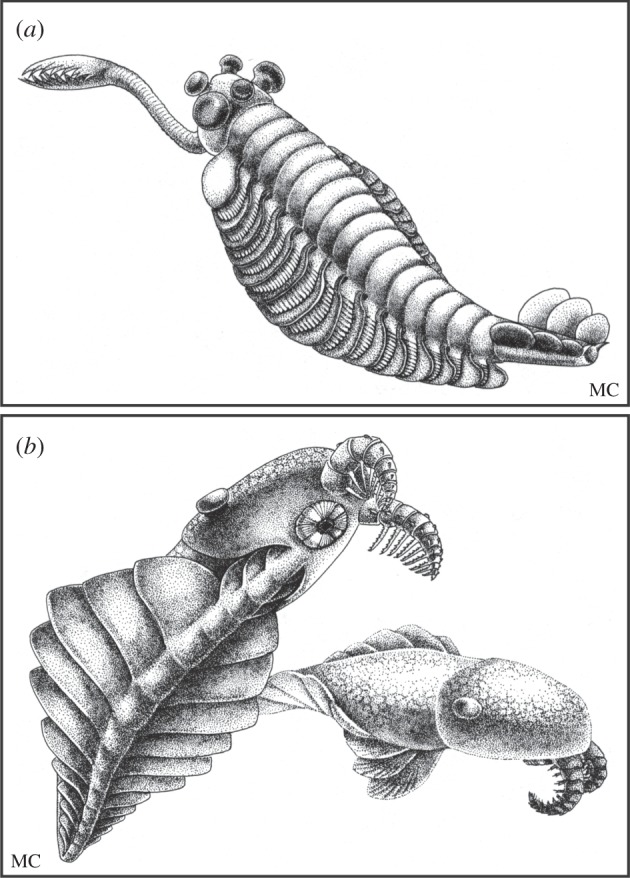
Illustrations of (*a*) *Opabinia* and (*b*) *Anomalocaris* from Stephen J. Gould's *Wonderful life* [6, figs 3.21 and 3.66, respectively]. © Marianne Collins, artist.

Manton's lifetime of investigations of the comparative functional morphology of living arthropods, together with the results of research by the Australian embryologist Don Anderson on arthropod development, led her to espouse a polyphyletic origin of the arthropods [20,21]. Whittington's reconstruction of *Opabinia* lent itself to an interpretation within this framework, which he had absorbed through reading Manton's work and discussing her ideas with her: the concept of separate phyla was a logical extension of Manton's view. In her 1973 synthesis, Manton [20] argued that Crustacea and Chelicerata evolved independently and not from annelids resembling those of today. She considered that Uniramia (arthropods with a single branched limb—insects, centipedes and millipedes, together with onychophorans) may have evolved from a segmented ancestor of annelids [19]. Figure 4 of her 1973 paper shows the major arthropod lineages evolving in parallel and not from a common ancestor. This also became Whittington's favoured hypothesis (figure 3) as shown in his presentation to the 1979 Systematics Association symposium on the *Origin of major invertebrate groups* [22]. Manton noted, without irony, that this pattern had been described as a phylogenetic lawn [21, p. 281], but R. P. S. (Dick) Jefferies remarked that ‘he thought it was terrible that Whittington and his group had presented an interpretation requiring either agnosticism or Special Creation’ [23, p. 488]!

## 7. An idea popularized

While Whittington's contradiction of Walcott's (and others) views on *Opabinia* were being published his graduate student Simon Conway Morris FRS was describing a selection of Burgess Shale animals that Walcott had overlooked: *Nectocaris*, *Odontogriphus*, *Dinomischus* and *Hallucigenia*. These too were difficult to ally with any living group. *Opabinia* was the first, but these other taxa also raised the possibility that taxa of high rank had evolved during the explosion of diversity that characterized the Cambrian, only to go extinct. *Opabinia*'s iconic status rests not only on its remarkable morphology, but the fact that it was the first newly described Burgess Shale animal that apparently could not be assigned to a living phylum. This concept of ‘taxa of uncertain affinity’ was seized upon by Gould [6] in his best selling *Wonderful life*, where he characterized them as ‘weird wonders’. The giant predator *Anomalocaris* (figure 4*b*), which Whittington and Briggs described in 1985 [24], was also included in this category. Gould saw Whittington's *Opabinia* paper as representing a conceptual leap in our understanding of the Cambrian explosion—tangible evidence that Burgess Shale taxa might represent a riot of experimental morphologies that are not represented today [6]. Gould [6, p. 136] believed that ‘Whittington's reconstruction of *Opabinia* … will stand as one of the great documents in the history of human knowledge’. Hyperbole surely but not, perhaps, if Whittington's *Opabinia* paper is viewed as a proxy for the Burgess Shale project that he led.

Whittington's fundamental insight led to a reconsideration of the relationships and significance of the Burgess Shale animals. It was Gould's *Wonderful life* [6], however, that catapulted them into the limelight and gave them popular substance through Marianne Collins' new renderings (figure 4) of the scientific reconstructions that Whittington and his team had published. Gould extended the concept of ‘weird wonders’ into a thesis about the evolution of morphology. He argued that the range of form (known as disparity, to distinguish it from diversity or number of taxa) that evolved in the Cambrian was much greater than at any time since, an idea based largely on the remarkable morphologies of creatures like *Opabinia*. Gould's book inspired attempts to quantify morphology, independently of numbers of taxa, and to determine how much variation appeared and how quickly during the Cambrian. Such analyses showed that disparity evolved rapidly in the Cambrian but to an amount that was not significantly greater than at any time later in the history of life [25]. In the meantime, new evidence showed that some of Gould's weird wonders were less remarkable than originally thought [26]. *Hallucigenia*, for example, turned out to be a spiny lobopod. At the same time, the use of cladistic methods for determining the relationships of fossils, which palaeontologists had been slow to adopt, was on the rise, and such an approach would ultimately help to clarify the relationships of Cambrian animals to those of today.

## 8. A different approach to determining relationships

Cladistics uses shared derived characters as a key to relationships between taxa, whereas considerations of disparity emphasized differences and, by implication, ‘weirdness’ compared with the more familiar creatures of today. As long as Whittington's team dwelt on the peculiarities of Burgess Shale animals like *Opabinia*, the affinities of these extinct forms were likely to be obscured. Whittington was not convinced of the value of numerical methods, much less cladistics, relying instead on experience and a deep knowledge of morphology to identify species and genera and determine their systematic position. In this, he was not unusual among trilobite specialists or other palaeontologists of the time. The validity of genera relied on their establishment by an expert on the group, and taxonomy was based, for the most part, on authority. The first attempt to use statistical methods to analyse the relationships of Burgess Shale organisms was a collaboration between Whittington and me, as one of his former graduate students [18,27]. We prepared a matrix of morphological data on 21 Cambrian arthropods and, with the help of John Temple, professor at Birkbeck College, University of London, an expert on the application of numerical taxonomy to trilobites, we used punched Fortran cards to carry out a principal components analysis which identified nearest neighbours based on all the morphological characters we coded. We used the same data to create a cladogram, but the result was not based on a computer algorithm but on our own assessment of the sequence in which shared characters evolved. This, Whittington's only foray into cladistics, was published in 1981 in the proceedings of the second international symposium on the Cambrian system held in Golden, Colorado [27]. The result, which separated bivalved taxa from those with a head shield, showed that Størmer's Trilobitomorpha was an artificial taxon. It was echoed by my assessment of the early radiation and relationships of the major arthropod groups with Richard Fortey FRS, in which we used a computer-based parsimony analysis (PAUP) [28]. Thus, there was an early acknowledgement, contra Gould, that the Burgess Shale arthropods, at least, were not polyphyletic, and that the Cambrian forms could be integrated into a phylogeny with those of today.

**Figure 5.**
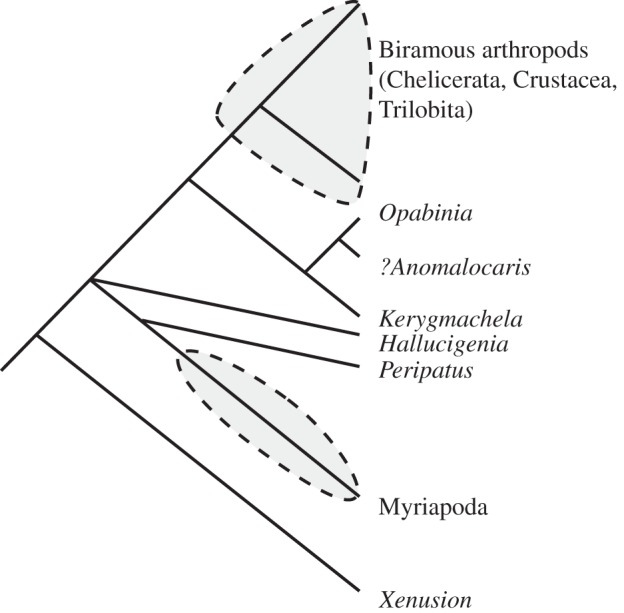
The first published cladogram to include *Opabinia* (adapted from [32, fig. 4]). *Opabinia*, *Kerygmachela*, and possibly *Anomalocaris* are grouped as a stem clade to the other arthropods. The enclosed clades are those that have become ‘arthropodized’, i.e. evolved a stiffened cuticle.

**Figure 6.**
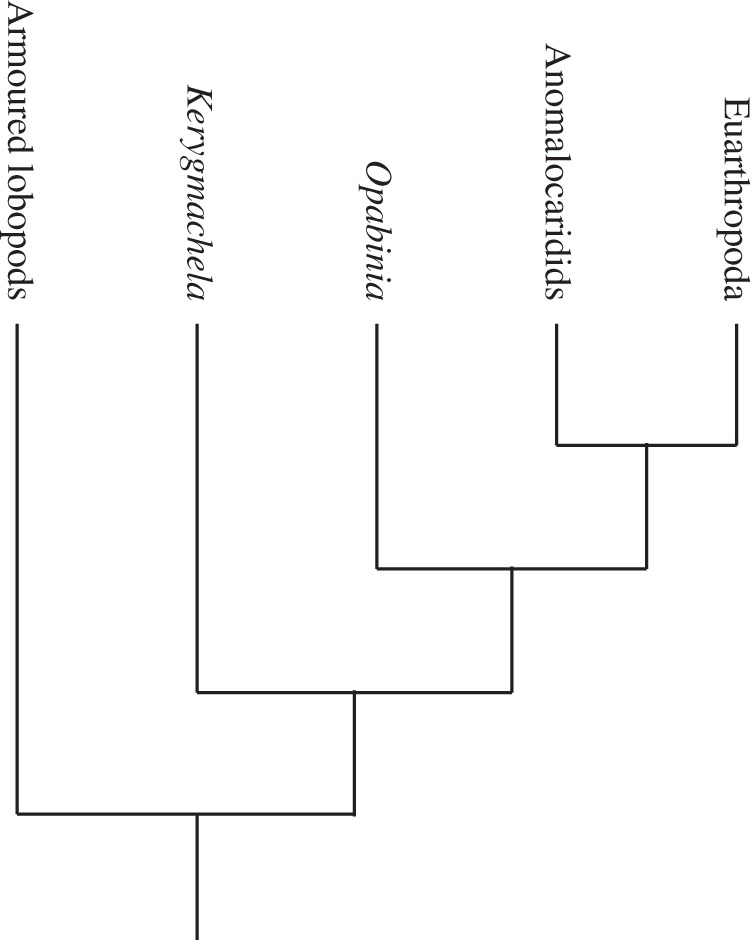
Present consensus on the position of *Opabinia* in the arthropod stem. (Adapted from [35, fig. 8], after [33–35].)

Notably, neither of these early applications of cladistics to the Burgess Shale arthropods included *Opabinia* or *Anomalocaris*. In 1987, Briggs & Whittington [29] were still arguing (contra Bergström [30–31]) that there was no evidence to ally *Opabinia* (or even *Anomalocaris*) with the arthropods. Graham Budd, Professor at the University of Uppsala in Sweden, started his career as a graduate student of Simon Conway Morris, working on another exceptionally preserved Cambrian fauna, from the Lower Cambrian of Sirius Passet, Greenland. He described a remarkable new arthropod, *Kerygmachela*, with lobe-like limbs and gill-like structures [32,33], drawing a comparison between the latter and the appendages of *Opabinia*. He presented the first cladogram showing these animals (figure 5), with the possible addition of *Anomalocaris*, forming a sister group to the biramous-limbed arthropods (Chelicerata, Crustacea, Trilobita), a *de facto* acknowledgement that *Opabinia* is an arthropod. Budd formalized this idea in 1996, interpreting *Opabinia* as having lobe limbs and placing it [34] as an offshoot of the stem just below *Anomalocaris*. This is not so far removed from Whittington's pre-cladistic view that *Opabinia* is representative of an ‘ancestral group of segmented animals' [1, p. 41] leading to modern arthropods. Although Budd's first cladograms [32,34] were not based on a parsimony analysis, they mark an important step forward in integrating Burgess Shale taxa into phylogenies with living organisms, using the concept of stem groups [18]. Budd's later parsimony analysis [33] represents the current consensus [35] on the position that *Opabinia* occupies (figure 6), although there remain controversies about its morphology.

## 9. Morphological debates

The flaps along the trunk of *Opabinia* were difficult to interpret from the outset. Walcott considered them limbs, but Whittington, well versed in trilobites and other arthropods, wondered if they might be pleurae (manuscript notes and correspondence with Manton in archives of Sedgwick Museum of Earth Sciences, University of Cambridge, ref.: WHTN Boxes 17 and 33). He eventually determined that the flaps were appendages, but unusual in having separate gill filaments on their outer surface (filaments are an integral part of such a flap in most other Cambrian arthropods although a similar arrangement to that in Whittington's reconstruction of *Opabinia* has recently been reported in anomalocaridids: [36]). This unusual arrangement may have been influenced by his initial interpretation of the flaps as pleurae, which required that they were not related to the gills in any way. Whittington had difficulty in deciding whether the first gill was associated with the first or the second flap (manuscript notes, ref.: WHTN Box 17) but decided that an external gill was absent on the first flap. Budd, prompted by the position of *Opabinia* on the arthropod stem between lobopods (such as *Aysheaia*) and Euarthopoda, went in search of the second limb branch (endopodite) [34] that Whittington did not find [9, p. 71]. Budd posited that lobe limbs would be present rather than jointed limbs and argued (figure 2*d*) that they are represented by the triangular reflective areas that Whittington interpreted as gut extensions. Zhang & Briggs [35] showed that the evidence presented by Budd for lobe limbs in *Opabinia* was equivocal and interpreted the gill filaments as extensions of the rear margin of the flap immediately to the anterior, resulting in a more familiar arthropod limb structure (figure 2*e*). More importantly, Zhang & Briggs agreed with Budd's placement of *Opabinia* on the arthropod stem [33,34], which does not vary regardless of whether lobe limbs were present or not (figure 6).

Ever tenacious, Budd, with his then graduate student Alison Daley, investigated *Opabinia* further [37], including new evidence from specimens in the Smithsonian and the Royal Ontario Museum. Their new interpretation, which largely supports Budd's reconstruction [34] (figure 2*d*), returned to Whittington's view that the gill-like structures are attached to the flap-like limbs dorsally (a position homologous with the gills of anomalocaridids) [1]. They also presented new evidence for the presence of lobopods. This is not the place to review the differences of interpretation in detail. Daley and Budd's evidence that setal-bearing margins are not present on the flaps (contra Zhang & Briggs [35]) may reflect the more distal outline of these structures, which does lack setae, and the outline of the supposed lobopod limbs is obscured by flattening onto other structures. The ongoing controversy about the morphology of *Opabinia* emphasizes the potential importance of this animal in determining the sequence of events in the evolution of arthropod limbs. At the same time, *Opabinia* has also captured the imagination of the public. A Google search for *Opabinia* images yields artwork, cartoons, T-shirts, jewellery and even soft toys—impressive, even if only a fraction of the number of hits for *Tyrannosaurus*!

## 10. The importance of exceptional preservation

The reinvestigation of the Burgess Shale under Whittington's leadership emphasized the importance of exceptional deposits in preserving soft-bodied animals in addition to the shelly forms that make up the normal fossil record. Whittington highlighted this by organizing a landmark meeting at the Royal Society in 1985, with Conway Morris, on *Extraordinary fossil biotas: their ecological and evolutionary significance* [38]. Conway Morris [39] performed a census of the collections in the Smithsonian documenting the proportions of different taxa and modes of life in the fauna as a whole. He showed that only about 14% of Burgess Shale genera and perhaps only 2% of individuals would be represented in the normal Cambrian fossil record. And many of the soft-bodied animals, like *Opabinia*, reveal combinations of characters that are not represented in the modern biota. The biota today is a winnowed sample—many of the intermediate forms have suffered extinction. These extinct intermediates are critical to unravelling how morphology evolves, and in analysing the relationships of even the modern groups. The Burgess Shale project stimulated huge interest in the evolutionary significance of exceptionally preserved fossils, which occur in what are commonly known as Konservat–Lagerstätten (conservation deposits). Erwin *et al*. [40] enumerated 33 phyla of which 19 are primarily or exclusively soft-bodied. Molecular phylogenies indicate that all 19 had evolved by the Cambrian and most of them (14) are already known from the Cambrian fossil record. The five that are not (rotifers, platyhelminthes, nematodes, entoprocts and nemerteans) are very small and delicate, with a very low fossilization potential.

One of Whittington's first tasks was to work out how the Burgess Shale fossils are flattened in the shale, a prerequisite to interpreting and reconstructing them. Much effort has subsequently been expended in attempts to explain why the fauna as a whole is preserved and, specifically, why Konservat–Lagerstätten are abundant in the Cambrian relative to later periods of time. Burgess Shale-type fossils are preserved as carbonaceous films [41,42]; decay-prone structures such as arthropod guts are sometimes preserved by very early replication in minerals such as apatite. We now know from analyses of the geochemistry of a range of Burgess Shale-type deposits that the chemistry of the Cambrian oceans was unusual [43]. The concentration of sulfate was low which may have somewhat inhibited decay by sulfate-reducing bacteria. At the same time, alkalinity was high leading to early carbonate cementation that sealed the sediment on the ocean floor. This combination was global in its influence and provides an explanation for the survival of carbonaceous remains and the concentration of exceptionally preserved fossil deposits in Cambrian rocks [43]. Although Whittington did not know it, conditions during the Cambrian favoured the preservation of soft-bodied fossils enhancing the available evidence for the nature of the Cambrian explosion.

## 11. New Burgess Shale-type deposits

When Whittington described *Opabinia*, the Burgess Shale was by far the most important repository of data on soft-bodied forms from the Cambrian. Although *Opabinia* remains essentially unique to the Walcott Quarry, a number of new discoveries have significantly augmented the number of Konservat–Lagerstätten of Cambrian age, and increased our knowledge of the diversity of Cambrian life. The immediate area around the Walcott Quarry has yielded several new sites of exceptional preservation. A number were discovered in the early 1980s by Royal Ontario Museum expeditions led by Desmond Collins [44], and the ROM has continued to play a central role in exploration in recent years under the direction of his successor Jean-Bernard Caron. A new locality at Stanley Glacier was reported in 2010 [45] and a slightly younger one at Marble Canyon [46], some 40 km from the original Walcott Quarry, was reported in 2014 and is under excavation. Marble Canyon has already yielded remarkable new specimens of the Burgess Shale chordate *Metaspriggina* [47]. Such new discoveries have shown that exceptional preservation in the Cambrian is much more widespread than we first realized.

In addition to new discoveries in the area around Walcott's original quarry, many important sites yielding Burgess Shale-type fossils have turned up in other parts of the world and are the subject of major research projects. A number of sites in the USA, notably in Utah, continue to yield new soft-bodied fossils, but they are generally not as well preserved as those from the original Burgess Shale. Spectacular Cambrian fossils have been found in China [48], particularly in the Cambrian of Yunnan Province, e.g. the Chengjiang and Guanshan biotas, but also in the middle Cambrian Kaili biota. There were hints in the older literature that Cambrian soft-bodied fossils might await discovery in Yunnan Province but modern work on the early Cambrian soft-bodied Chengjiang biota, in the Maotianshan Shales, dates from the discovery of a naraoiid arthropod with limbs by Chinese palaeontologist Hou Xian-Guang in 1984 [49]. Chengjiang, which rivals the original Burgess Shale in importance, now embraces a number of Konservat–Lagerstätten in much the same way that there are several in the area of Walcott's Quarry. Several other Burgess Shale-type biotas have been discovered or come on stream since Whittington initiated his project. Sirius Passet, in the Lower Cambrian of Greenland, was discovered in 1984 [50] and is the source, most notably, of the armoured slug-like creature *Halkieria* [51]. The Lower Cambrian Emu Bay Shale on the coast of Kangaroo Island in South Australia was reported by Martin Glaessner in 1979 but is now the site of new excavations inland and has yielded highlights such as anomalocaridid eyes [52]. Briggs & Nedin [53] even suggested that *Myoscolex*, one of the more common fossils from the Emu Bay Shale, might be *Opabinia*-like. The majority of specimens of *Myoscolex*, however, consist of phosphatized muscle blocks—the cuticle, or evidence of appendages, is rarely preserved and the nature of *Myoscolex* has yet to be confirmed. More recently, Van Roy *et al*. [54] reported a Burgess Shale-type fauna with iconic Burgess Shale taxa such as marrellomorphs and giant complete anomalocaridids from the Ordovician of Morocco. When Whittington was describing *Opabinia* in 1975, such palaeontological riches were inconceivable.

## 12. The next step

Future work on the Cambrian will involve exploiting the wealth of new fossil discoveries around the world. The approach, however, is increasingly interdisciplinary. Refined methods of imaging fossils, reconstructing the original animal, analysing relationships and generating phylogenies, and dating and analysing rock sequences, open up remarkable possibilities for working out the early evolution of animals and relating it to environment and ecology. The most exciting progress, however, will come from an integration of palaeontological and biological approaches. Molecular phylogenies combined with fossil data provide a new measure of rates and steps in the evolution of clades. And relating fossil morphologies to studies of evolutionary development and how it is controlled genetically [55] will reveal mechanisms for the rapid morphological change that characterizes the Cambrian. One day, we will have a much more complete understanding of the evolutionary processes that link *Opabinia* with lobopods like *Hallucigenia*, anomalocaridids and the arthropods of today.

## Author profile

**Derek E. G. Briggs** is G. Evelyn Hutchinson Professor of Geology and Geophysics at Yale University. His primary research interest is in the preservation and evolutionary significance of exceptionally preserved fossils. He has been at Yale University since 2003 where he has served successively as director of the Yale Institute for Biospheric Studies and the Yale Peabody Museum of Natural History. Prior to joining Yale, Briggs was at the University of Bristol for 18 years following 8 years at Goldsmiths, University of London. Briggs is a graduate of Trinity College, Dublin, and studied arthropods from the Burgess Shale at Cambridge University with Harry Whittington for his PhD. Briggs was elected a Fellow of the Royal Society in 1999 and is an honorary Member of the Royal Irish Academy. He has published more than 300 scientific articles and several books including *The fossils of the Burgess Shale* (with D. H. Erwin and F. J. Collier: Smithsonian Press, 1994). (Photo by J.O. Briggs.)

## References

[1] Whittington HB 1975 The enigmatic animal *Opabinia regalis*, Middle Cambrian, Burgess Shale, British Columbia. Phil. Trans. R. Soc. Lond. B 271, 1–43. (10.1098/rstb.1975.0033) PMC4360120 25750235

[2] Briggs DEG 2010 Obituary: Harry Whittington (1916–2010). Nature 466, 706 (10.1038/466706a) 20686564

[3] Fortey RA 2012 Harry Blackmore Whittington. 24 March 1916–20 June 2010. Biogr. Mems. Fell. R. Soc. 58, 299–325. (10.1098/rsbm.2012.0033)

[4] Conway Morris S 2014 Whittington, Harry Blackmore (1916–2010). In Oxford dictionary of national biography (online). Oxford, UK: Oxford University Press (10.1093/ref:odnb/103342)

[5] Walcott CD 1918 Appendages of trilobites. Cambrian geology and paleontology, IV. Smithsonian Misc. Coll. 67, 115–216.

[6] Gould SJ 1989 Wonderful life. The Burgess Shale and the nature of history. New York, NY: W.W. Norton and Company.

[7] Rolfe WDI 1963 Catalogue of type specimens in the invertebrate paleontological collections of the Museum of Comparative Zoology Arthropoda (Trilobita, Arachnida and Insecta excluded). Bull. Mus. Comp. Zool. 129, 369–398.

[8] Rolfe WDI 1962 Two new arthropod carapaces from the Burgess Shale (Middle Cambrian) of Canada. Breviora 160, 1–9.

[9] Whittington HB 1985 The Burgess Shale. New Haven, CT: Yale University Press.

[10] Størmer L 1959 Trilobitoidea. In Treatise on invertebrate paleontology, part O, Arthropoda (ed. Moore RC), pp. 23–37. Lawrence, KS: Geological Society of America and University of Kansas Press.

[11] Whittington HB 1971 Redescription of *Marrella splendens* (Trilobitoidea) from the Burgess Shale, Middle Cambrian, British Columbia. Bull. Geol. Surv. Can. 209, 1–24. (10.4095/102427)

[12] Whittington HB 1971 The Burgess Shale: history of research and preservation of fossils. In Proc. North American Paleontological Convention, Chicago 1969, Part I, pp. 1170–1201. Lawrence, KS: Allen Press.

[13] Whittington HB 1974 *Yohoia* Walcott and *Plenocaris* n. gen., arthropods from the Burgess Shale, Middle Cambrian, British Columbia*.* Bull. Geol. Surv. Can. 231, 1–21.

[14] Whittington HB 1975 Trilobites with appendages from the Middle Cambrian Burgess Shale, British Columbia. Fossils Strata 4, 97–136.

[15] Walcott CD 1912 Middle Cambrian Branchiopoda, Malacostraca, Trilobita and Merostomata. Cambrian geology and paleontology, II. Smithsonian Misc. Coll. 57, 146–228.

[16] Hutchinson GE 1930 Restudy of some Burgess Shale fossils. Proc. US Nat. Mus. 78, 1–11. (10.5479/si.00963801.78-2854.1)

[17] Simonetta AM 1970 Studies on non trilobite arthropods of the Burgess Shale (Middle Cambrian). Palaeontogr. Ital. 66, 35–45.

[18] Brysse K 2008 From weird wonders to stem lineages: the second reclassification of the Burgess Shale fauna. Stud. Hist. Philos. Biomed. Sci. 39, 298–313. (10.1016/j.shpsc.2008.06.004) 18761282

[19] Manton SM 1977 The Arthropoda: habits, functional morphology, and evolution. London, UK: Clarendon Press.

[20] Manton SM 1973 Arthropod phylogeny: a modern synthesis. J. Zool. Lond. 171, 111–130. (10.1111/j.1469-7998.1973.tb07519.x)

[21] Manton SM Anderson DT 1979 Polyphyly and the evolution of the arthropods. In The origin of major invertebrate groups. *Systematics Association special vol. 12* (ed. House MR), pp. 269–321. London, UK: Academic Press.

[22] Whittington HB 1979 Early arthropods, their appendages and relationships. In The origin of major invertebrate groups. *Systematics Association special vol. 12* (ed. House MR), pp. 253–268. London, UK: Academic Press.

[23] House MR 1979 Discussion on the origin of major invertebrate groups. In The origin of major invertebrate groups. *Systematics Association special vol. 12* (ed. House MR), pp. 479–494. London, UK: Academic Press.

[24] Whittington HB Briggs DEG 1985 The largest Cambrian animal, *Anomalocaris*, Burgess Shale, British Columbia. Phil. Trans. R. Soc. Lond. B 309, 569–609. (10.1098/rstb.1985.0096)

[25] Wills MA Briggs DEG Fortey RA 1994 Disparity as an evolutionary index: a comparison of Cambrian and Recent arthropods. Paleobiology 20, 93–130. (10.2307/2401014)

[26] Briggs DEG Fortey RA 2005 Wonderful strife: systematics, stem groups, and the phylogenetic signal of the Cambrian radiation. Paleobiology 31 (Suppl. 2), 94–112. (10.1666/0094-8373(2005)031[0094:WSSSGA]2.0.CO;2)

[27] Briggs DEG Whittington HB 1981 Relationships of arthropods from the Burgess Shale and other Cambrian sequences. In Short papers for the second international symposium on the Cambrian system, pp. 38–41. US Department of the Interior: Geological Survey Open-File Report 22.

[28] Briggs DEG Fortey RA 1989 The early radiation and relationships of the major arthropod groups. Science 246, 241–243. (10.1126/science.246.4927.241) 17839017

[29] Briggs DEG Whittington HB 1987 The affinities of the Cambrian animals *Anomalocaris* and *Opabinia*. Lethaia 20, 185–186. (10.1111/j.1502-3931.1987.tb02036.x)

[30] Bergström J 1986 *Opabinia* and *Anomalocaris*, unique Cambrian ‘arthropods’. Lethaia 19, 241–146. (10.1111/j.1502-3931.1986.tb00738.x)

[31] Bergström J 1987 The Cambrian *Opabinia* and *Anomalocaris*. Lethaia 20, 187–188. (10.1111/j.1502-3931.1987.tb02037.x)

[32] Budd GE 1993 A Cambrian gilled lobopod from Greenland. Nature 364, 709–711. (10.1038/364709a0)

[33] Budd GE 1999 The morphology and phylogenetic significance of *Kerygmachela kierkegaardi* Budd (Buen Formation, Lower Cambrian, N Greenland). Trans. R. Soc. Edinb. Earth Sci. 89, 249–290. (10.1017/S0263593300002418)

[34] Budd GE 1996 The morphology of *Opabinia regalis* and the reconstruction of the arthropod stem-group. Lethaia 29, 1–14. (10.1111/j.1502-3931.1996.tb01831.x)

[35] Zhang X-L Briggs DEG 2007 The nature and significance of the appendages of *Opabinia* from the Middle Cambrian Burgess Shale*.* Lethaia 40, 161–173. (10.1111/j.1502-3931.2007.00013.x)

[36] Daley AC Budd GE Caron J-B Edgecombe GD Collins D 2009 The Burgess Shale anomalocaridid *Hurdia* and its significance for early euarthropod evolution. Science 323, 1597–1600. (10.1126/science.1169514) 19299617

[37] Budd GE Daley AC 2012 The lobes and lobopods of *Opabinia regalis* from the middle Cambrian Burgess Shale. Lethaia 45, 83–95. (10.1111/j.1502-3931.2011.00264.x)

[38] Whittington HB Conway Morris S (eds). 1985 Extraordinary fossil biotas: their ecological and evolutionary significance. Proceedings of a Royal Society Discussion Meeting held on 20 and 21 February 1985. Phil. Trans. R. Soc. Lond. B 311, 1–192.

[39] Conway Morris S 1986 The community structure of the Middle Cambrian phyllopod bed (Burgess Shale). Palaeontology 29, 423–467.

[40] Erwin DH Laflamme M Tweedt SM Sperling EA Pisani D Peterson KJ 2011 The Cambrian conundrum: early divergence and later ecological success in the early history of animals. Science 334, 1091–1097. (10.1126/science.1206375) 22116879

[41] Butterfield NJ 1995 Secular distribution of Burgess Shale-type preservation. Lethaia 28, 1–13. (10.1111/j.1502–3931.1995.tb01587.x)

[42] Gaines RR Briggs DEG Zhao Y-L 2008 Cambrian Burgess Shale-type deposits share a common mode of fossilization. Geology 36, 755–758. (10.1130/G24961A.1)

[43] Gaines RR Hammarlund EU Hou X-G Qi C-S Gabbott SE Zhao Y-L Peng J Canfield DE 2012 Mechanism for Burgess Shale-type preservation. Proc. Natl Acad. Sci. USA 109, 5180–5184. (10.1073/pnas.1111784109) 22392974 PMC3325652

[44] Collins D Briggs DEG Conway Morris S 1983 New Burgess Shale fossil sites reveal Middle Cambrian faunal complex. Science 222, 163–167. (10.1126/science.222.4620.163.) 17741659

[45] Caron J-B Gaines RR Gabriela Mángano M Streng M Daley AC 2010 A new Burgess Shale-type assemblage from the ‘thin’ Stephen Formation of the southern Canadian Rockies. Geology 38, 811–814. (10.1130/G31080.1)

[46] Caron J-B Gaines RR Aria C Gabriela Mángano M Streng M 2014 A new phyllopod bed-like assemblage from the Burgess Shale of the Canadian Rockies. Nat. Commun. 5, 1–6. (10.1038/ncomms4210) 24513643

[47] Conway Morris S Caron J-B 2014 A primitive fish from the Cambrian of North America. Nature 512, 419–422. (10.1038/nature13414) 24919146

[48] Steiner M Zhu M-Y Zhao Y-L Erdtmann B-D 2005 Lower Cambrian Burgess Shale-type fossil associations of South China. Palaeogeogr. Palaeoclimatol. Palaeoecol. 220, 129–152. (10.1016/j.palaeo.2003.06.001)

[49] Hou X-G Aldridge RJ Bergström J Siveter DJ Siveter DJ Feng XH 2004 The Cambrian fossils of Chengjiang, China. Oxford, UK: Blackwell Publishing.

[50] Conway Morris S Peel JS Higgins AK Soper NJ Davis NC 1987 A Burgess Shale-like fauna from the Lower Cambrian of North Greenland. Nature 326, 181–183. (10.1038/326181a0)

[51] Conway Morris S Peel JS 1990 Articulated halkieriids from the Lower Cambrian of north Greenland. Nature 345, 802–805. (10.1038/345802a0)

[52] Paterson JR Garcia-Bellido DC Lee MSY Brock GA Jago JB Edgecombe GD 2011 Acute vision in the giant Cambrian predator *Anomalocaris* and the origin of compound eyes. Nature 480, 237–240. (10.1038/nature10689) 22158247

[53] Briggs DEG Nedin C 1997 The taphonomy and affinities of the problematic fossil *Myoscolex* from the Lower Cambrian Emu Bay Shale of South Australia. J. Paleontol. 71, 22–32.

[54] Van Roy P Orr PJ Botting JP Muir LA Vinther J Lefebvre B el Hariri K Briggs DEG 2010 Ordovician faunas of Burgess Shale type. Nature 465, 215–218. (10.1038/nature09038) 20463737

[55] Briggs DEG Siveter DJ Siveter DJ Sutton MD Garwood RJ Legg D 2012 Silurian horseshoe crab illuminates the evolution of chelicerate limbs. Proc. Natl Acad. Sci. USA 109, 15 702–15 705. (10.1073/pnas.1205875109) 22967511 PMC3465403

